# Circadian Clock Synchronization of the Cell Cycle in Zebrafish Occurs through a Gating Mechanism Rather Than a Period-phase Locking Process

**DOI:** 10.1177/0748730418755583

**Published:** 2018-02-14

**Authors:** Ricardo Laranjeiro, T. Katherine Tamai, William Letton, Noémie Hamilton, David Whitmore

**Affiliations:** Centre for Cell and Molecular Dynamics, Department of Cell and Developmental Biology, University College London, London, UK; 1. Department of Molecular Biology and Biochemistry, Nelson Biological Laboratories, Rutgers, The State University of New Jersey, Piscataway, NJ 08854, USA; 2. Institute of Transformative Bio-Molecules (ITbM), Nagoya University, Nagoya 464-8601, Japan; 3. Bateson Centre, University of Sheffield, Firth Court, Western Bank, Sheffield, S10 2TN, UK

**Keywords:** cell cycle, entrainment, T-cycle, zebrafish, gating

## Abstract

Studies from a number of model systems have shown that the circadian clock controls expression of key cell cycle checkpoints, thus providing permissive or inhibitory windows in which specific cell cycle events can occur. However, a major question remains: Is the clock actually regulating the cell cycle through such a gating mechanism or, alternatively, is there a coupling process that controls the speed of cell cycle progression? Using our light-responsive zebrafish cell lines, we address this issue directly by synchronizing the cell cycle in culture simply by changing the entraining light-dark (LD) cycle in the incubator without the need for pharmacological intervention. Our results show that the cell cycle rapidly reentrains to a shifted LD cycle within 36 h, with changes in *p21* expression and subsequent S phase timing occurring within the first few hours of resetting. Reentrainment of mitosis appears to lag S phase resetting by 1 circadian cycle. The range of entrainment of the zebrafish clock to differing LD cycles is large, from 16 to 32 hour periods. We exploited this feature to explore cell cycle entrainment at both the population and single cell levels. At the population level, cell cycle length is shortened or lengthened under corresponding T-cycles, suggesting that a 1:1 coupling mechanism is capable of either speeding up or slowing down the cell cycle. However, analysis at the single cell level reveals that this, in fact, is not true and that a gating mechanism is the fundamental method of timed cell cycle regulation in zebrafish. Cell cycle length at the single cell level is virtually unaltered with varying T-cycles.

The central role of the circadian clock is to control the timing or phase of downstream cell biological, physiological, and behavioral processes. One of the most significant of these rhythmic outputs is the temporal control of the cell cycle in healthy, proliferative tissues. It is well-established that disruption of clock function leads to a loss of timed cell cycle events, with a consequent increase in cancer risk and tumor proliferation rates (Savvidis and Koutsilieris, 2012). How the circadian clock couples to the cell cycle is, therefore, of considerable importance, at both mechanistic and conceptual levels.

Several studies have identified key cell cycle regulators that are under direct control of the circadian clock. These include roles for *wee1* and *Cyclin B1-Cdc2* kinase expression in regenerating mammalian liver (Matsuo et al., 2003) and *p21* in regulating hepatocyte proliferation (Grechez-Cassiau et al., 2008). In proliferative fibroblasts, the multifunctional nuclear protein NONO regulates the transcription of the cell cycle checkpoint protein p16-Ink4A in a PERIOD protein-dependent manner (Kowalska et al., 2013). In zebrafish, *cyclin B1* expression rhythms have been implicated in regulating mitotic timing, whereas *p21* and the related *p20* gene appear to be essential for the clock regulation of DNA replication, or S phase timing (Tamai et al., 2012; Laranjeiro et al., 2013). All of these results point to the idea that the clock directly regulates well-established cell cycle checkpoint pathways and, in this way, establishes a “circadian checkpoint” mechanism for temporal cell cycle control. Such results imply that the clock uses these circadian checkpoints to create a window or gate that is either permissive or repressive for cell cycle progression. But is the clock actually coupling to the cell cycle through such a gating mechanism?

There are two general conceptual ways in which clock-cell cycle coupling could occur. One possibility is that the speed of progression, or angular velocity, of the cell cycle could be adjusted directly by the clock, such that the 2 periods become equivalent. Such a coupling mechanism might make sense for proliferative cells where the cell cycle length is close to 24 h, as in many cell types, and coincidentally falls within the “range of entrainment” of the circadian clock. Such 1:1 phase locking has been demonstrated in some mammalian proliferative cells, in particular NIH/3T3 mouse fibroblasts, by imaging both cell cycle progression and circadian clock gene expression rhythms in single cells (Bieler et al., 2014; Feillet et al., 2014). However, complexities in this 1:1 coupling are seen when the cellular circadian clock is synchronized by an external stimulus, producing several peaks in cell division (Matsuo et al., 2003; Feillet et al., 2014). An alternative model is that the timing of specific cell cycle events is restricted by a gating mechanism, in which the clock imposes a specific circadian checkpoint mechanism and subsequent phase on the cell cycle. Such a mechanism has been shown to exist in cyanobacteria (Mori et al., 1996; Yang et al., 2010). A gating mechanism might be more applicable in cells or tissues where the cell cycle length deviates significantly from 24 h and the duration of the cell cycle cannot be easily altered to match the 24-h period of the circadian clock. The mechanistic data described above, where well-defined cell cycle checkpoint proteins are “co-opted” by the clock, might also support the existence of a gating mechanism rather than a process that alters the “speed” of cell cycle progression in a continuous manner.

In this study, we aim to explore the issue of cell cycle entrainment using our zebrafish cell line system. These cells have the distinct advantage over mammalian cultures in that zebrafish cells are themselves light-sensitive, and, consequently, the clock can be entrained in culture by a biologically relevant stimulus (light), as opposed to an artificial, pharmacological one (forskolin or dexamethasone). We have previously shown that exposing cells to an LD cycle in culture not only sets the clock but also synchronizes the cell cycle as a downstream rhythmic output of the cellular clock system (Dekens et al., 2003; Tamai et al., 2012). The same circadian-cell cycle regulation occurs in developing embryos, in adult tissues such as the gut, and in zebrafish melanomas (Dekens et al., 2003; Dickmeis et al., 2007; Tamai et al., 2012; Laranjeiro et al., 2013; Peyric et al., 2013; Hamilton et al., 2015). By simply shifting the LD cycle in the incubator, we can determine how quickly the cell cycle adjusts to the new lighting regime in cell culture and identify the initial molecular changes that occur in the cell cycle mechanism following this phase shift in the clock. Using a T-cycle lighting regime, we can explore further not only the range of entrainment of the circadian clock but also how cell cycle length adjusts to this wide range of clock periods. Can cell cycle length shorten or lengthen to match a significant range of external driving cycles, and, if so, how much can it “change speed”? By analyzing both cell populations and single cells, we can determine directly whether a gating process or a 1:1 period-coupling mechanism is at work in zebrafish cells.

## Materials and Methods

### Cell Culture

Zebrafish cell lines were cultured in Leibovitz’s L-15 medium (Gibco, Gaithersburg, MD, USA) containing 15% fetal bovine serum (Biochrom AG, Berlin, Germany), 50 U/mL penicillin/streptomycin (Gibco, Gaithersburg, MD, USA), and 50 µg/mL gentamicin (Gibco). Cells were incubated in a large-volume, thermostatically controlled water bath at 28 °C on different LD cycles for the indicated time. Fluorescent desk lamps were used for light entrainment.

### Bioluminescence Assays

The luminescent reporter cell lines used in this study have been previously described: *Per1*-*luciferase* cells (Vallone et al., 2004), *p21(3 kb)*-*luciferase* and *p20(3.6 kb)*-*luciferase* cells (Laranjeiro et al., 2013), and *Cyclin B1*-*luciferase* cells (Tamai et al., 2012). Luminescent cell lines were plated (50,000-100,000 cells/well) in quadruplicate wells of a white 96-well plate (Greiner, Kremsmunster, Austria) in medium supplemented with 0.5 mM beetle luciferin (Promega, Madison, WI, USA). Plates were sealed with clear adhesive TopSeal (Perkin Elmer, Waltham, MA, USA). Bioluminescence was monitored on a TopCount NXT scintillation counter (Packard Instrument Company, Meriden, CT, USA). The 96-well plates were placed in a temperature-controlled chamber (~28 °C) under different lighting conditions. Approximately every hour, each plate was automatically taken into the dark counting chamber where luminescence in each well was measured as counts per second (CPS). These measurements took approximately 10 min for each 96-well plate, after which the plate was returned to the experimental lighting conditions.

The peak of expression of the various luminescent cell lines was first calculated in ZT. Then, the ZT was divided by the respective day length to obtain a percentage, corresponding to the relative peak of expression. For example, if the expression peak of a gene occurs at ZT3 in a 24-h LD cycle, the relative peak of expression is 3/24 = 0.125 = 12.5% of the day. To determine the relative time of day for each expression peak, the LD periods were divided as follows: early light phase (0%-18.75% of the day), middle light phase (18.76%-31.25% of the day), late light phase (31.26%-50% of the day), early dark phase (50.01%-68.75% of the day), middle dark phase (68.76%-81.25% of the day), and late dark phase (81.26%-100% of the day).

### BrdU Pulsing and Labeling of Cell Lines

Zebrafish PAC2 cells (Whitmore et al., 2000) were plated in triplicate wells of a 6-well dish (Greiner) and pulsed at the indicated ZT with 10 µM BrdU for 30 min at 28 °C. Immediately after BrdU exposure, cells were dissociated with 0.05% trypsin-EDTA (Gibco) and fixed in cold 70% ethanol overnight at 4 °C. After washes in PBS, cells were treated with 2 M HCl for 30 min at room temperature, followed by washes in PBS and PBS, 0.2% Tween 20, 0.1% bovine serum albumin (PBSTB). Cell pellets were incubated directly with 2 µL of mouse anti-BrdU antibody (BD Biosciences, Franklin Lakes, NJ, USA) for 20 min at room temperature. After a wash in PBSTB, cells were incubated with Cy5-conjugated, goat anti-mouse antibody (1:10, Molecular Probes, Eugene, OR, USA) in PBSTB for 20 min at room temperature. Finally, cells were washed in PBS, followed by treatment with 100 µg/mL RNase A (Sigma-Aldrich, St. Louis, MO, USA) and staining with 50 µg/mL propidium iodide (Sigma-Aldrich, St. Louis, MO, USA). Samples were then analyzed by flow cytometry on a FACSCalibur (BD Biosciences) in the FACS facility at Cancer Research UK London Research Institute, and data were analyzed using FlowJo software (Tree Star Inc., San Carlos, CA, USA).

### Cell Proliferation Assays

For cell proliferation assays, zebrafish PAC2 cells were plated in triplicate wells of a 6-well dish at 50,000 cells per well (low density) or 400,000 cells per well (high density). Quantification of the total number of cells was performed by treating cell cultures with 0.05% trypsin-EDTA until a single cell suspension was obtained, followed by cell counting with a hemocytometer.

### Time-lapse Imaging of Zebrafish FUCCI Cells

The zebrafish FUCCI cell line used in this study has been previously described (Downey et al., 2011). FUCCI cells were mixed with nonfluorescent PAC2 cells at a ratio between 1:25 and 1:50 to allow tracking of individual cells in a confluent cell culture. These mixed cultures were incubated in a water bath at 28 °C for approximately 96 h under an entraining T-cycle. Cells were then imaged on an ImageXpress Micro XL Widefield High Content Screening System (Molecular Devices, Sunnyvale, CA, USA) every 40 min for 72 h. Temperature and LD cycle were maintained during time-lapse recording. Single cell tracking was performed manually in ImageJ.

### Statistical Analysis

The data in this study are presented as the average ± standard error of the mean (*n* ≥ 3). Statistical significance was determined by 1-way ANOVA followed by Tukey’s post hoc test when necessary. *P* values less than 0.05 were considered statistically significant.

## Results

### Zebrafish Cell Cycle Rhythms Are Rapidly Reentrained to a Reverse LD Cycle

Although we know a considerable amount about circadian clock entrainment in zebrafish, very little is known about how the cell cycle becomes entrained to an environmental LD cycle. Initially, we asked whether circadian cell cycle timing can be reentrained to a reverse light-dark cycle (DL) and, if so, how rapidly does reentrainment occur? To address these questions, we pulsed zebrafish cell cultures with BrdU to determine the number of cells in S phase at any given time of day. We then used zebrafish luminescent reporter cell lines for the cell cycle regulators *p21* and *Cyclin B1. p21* regulates the G1/S transition in zebrafish cell lines and is a direct clock target gene (Laranjeiro et al., 2013). *Cyclin B1* is required for the G2/M transition of the cell cycle and has been shown previously to be rhythmically expressed in zebrafish cell lines, although there is currently no evidence that its transcription is directly clock-controlled (Tamai et al., 2012).

**Figure 1.**
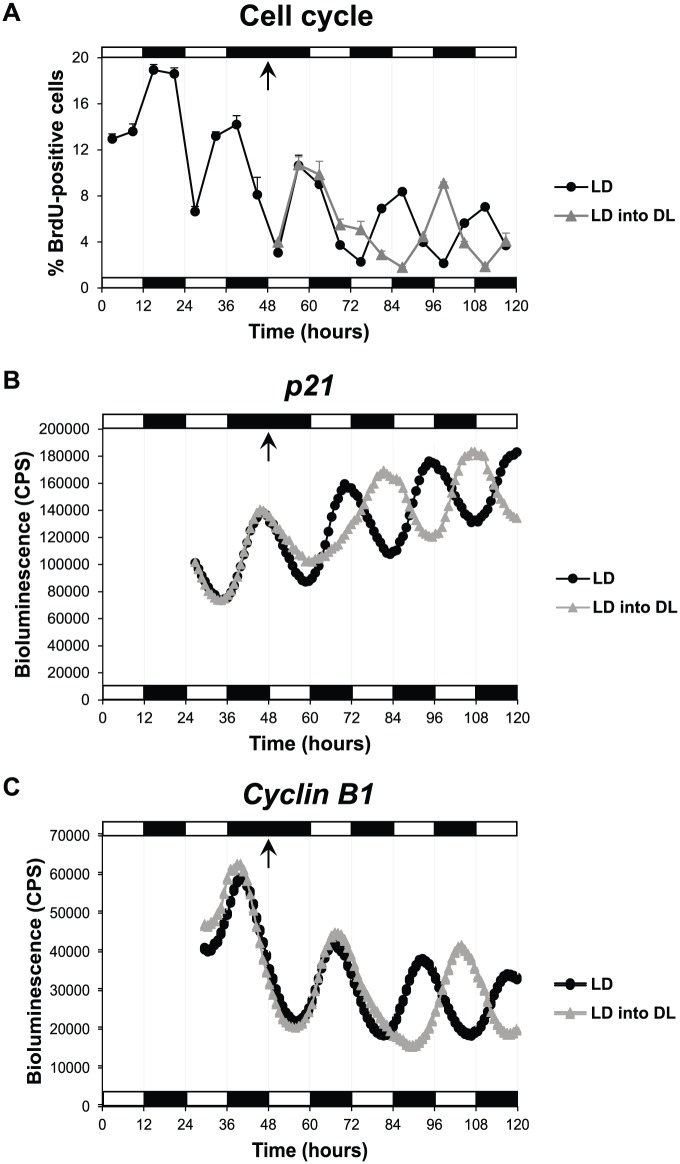
Zebrafish cell cycle rhythms are rapidly reentrained to a reverse LD cycle. (A) Percentage of PAC2 cells in S phase (BrdU-positive cells) when exposed to an LD cycle or to an LD into DL cycle. Bioluminescent traces of (B) *p21-luciferase* and (C) *Cyclin B1-luciferase* cell lines exposed to an LD cycle or an LD into DL cycle. Arrows indicate the transition point from an LD to DL cycle. White and black bars represent light and dark phases, respectively.

Following reversal of the light-dark cycle, it is clear that S phase rhythms exhibited the first signs of reentrainment to the new DL cycle within 24 h, with antiphasic entrainment complete by the end of the second day (Fig. 1A). Cell cycle reentrainment appears to proceed by an increase in the number of cells entering S phase, and so a likely “relaxation” of the G1-S cell cycle checkpoint occurs. To explore this phenomenon further, we reversed the LD cycle while monitoring expression of the major G1-S checkpoint regulatory gene, *p21*, dynamically in live cells. We found that the *p21* expression pattern changed almost immediately, with expression levels significantly higher just a few hours after shifting the LD cycle (Fig. 1B). This change led to a perfect antiphasic entrainment of *p21* expression within 36 h of the new lighting regime (Fig. 1B). In contrast, the first signs of a *Cyclin B1* reentrainment took more than 24 h to become evident (Fig. 1C). Complete antiphasic entrainment of *Cyclin B1* expression was observed by 48 h after light cycle reversal and was stable in the subsequent cycles (Fig. 1C). Thus, both cell cycle genes reentrained to the reverse light-dark cycle, but at clearly different rates.

Cell cycle reentrainment appears to occur with an initial increase in cells entering S phase. These S phase changes match precisely the rapid change we detect in *p21* expression. An initial decline in *p21* levels upon a change in lighting would allow more cells to move from G1 to S, followed by a strong inhibition of this event. Since *p21* is a direct clock target gene, our results suggest that the core circadian clock responds immediately to the DL cycle exposure and then transfers that new phase information to the cell cycle via a rapid alteration in *p21* expression. Changes in mitosis, however, require an additional circadian cycle to be complete.

### Clock-related Gene Expression Is Entrained to a Wide Range of LD Cycle Lengths

Given the impressive reentrainment rate of the zebrafish circadian clock and cell cycle timing, we sought to determine how flexible the circadian clock can be when exposed to zeitgeber cycles with a period (T) different from 24 h, T-cycles. We used multiple zebrafish luminescent reporter cell lines in these experiments: To study core clock entrainment, we monitored *Per1*-luciferase expression; to follow S phase timing and direct downstream clock target genes, we monitored both *p21-* and *p20*-luciferase expression; and to measure mitotic timing and an indirect clock target gene, we monitored *Cyclin B1*-luciferase expression.

**Figure 2.**
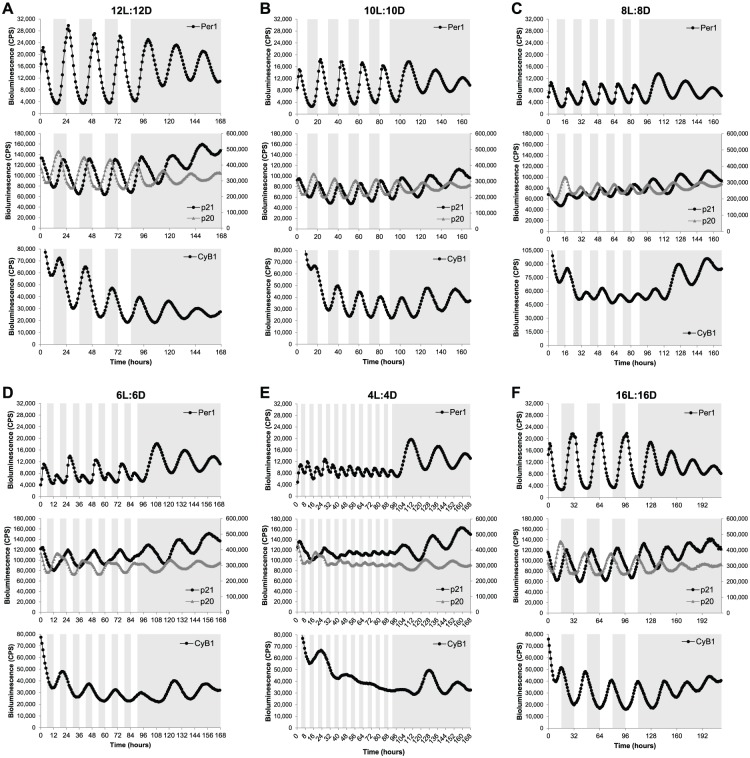
Clock-related gene expression is entrained to a wide range of LD cycles. Bioluminescent traces of *Per1*-, *p21*-, *p20*-, and *Cyclin B1*-*luciferase* cell lines exposed to (A) 12L:12D, (B) 10L:10D, (C) 8L:8D, (D) 6L:6D, (E) 4L:4D, or (F) 16L:16D cycles and then transferred to DD. Note that the peak of *Cyclin B1* expression in the 8L:8D cycle (C) is progressively delayed in each LD cycle (first peak at ZT2.6, second peak at ZT4.3, third peak at ZT4.4, fourth peak at ZT6.2, and fifth peak at ZT6.3). The left and right axes of the middle panels correspond to bioluminescence levels of *p21* and *p20* reporter cell lines, respectively. Note that *p21* and *p20* reporter cell lines were constructed with different *luciferase* genes; therefore, bioluminescence levels are not comparable. White and gray backgrounds represent light and dark phases, respectively.

Reporter cell lines were exposed to a normal LD cycle (12L:12D) for 4 complete cycles, followed by transfer into constant darkness (DD). As expected, all genes showed clear rhythmic expression in 12L:12D conditions, which were maintained in DD (Fig. 2A). We then tested shorter T-cycles, namely 10L:10D (T = 20) and 8L:8D (T = 16) cycles. Under these conditions, gene expression was entrained to the new period lengths, showing an expression peak every 20 h (Fig. 2B) or 16 h (Fig. 2C) during the LD cycle for 10L:10D and 8L:8D cycles, respectively. However, the amplitude of the expression rhythms was lower in the shorter T-cycles, particularly in 8L:8D, than in the normal 12L:12D cycle (Fig. 2, A-C). Interestingly, in the 8L:8D cycle, the peak of *Cyclin B1* expression was slightly delayed in every subsequent LD cycle compared with the previous one (progressive delay in expression peak from ZT2.6 on the first LD cycle to ZT6.3 on the last LD cycle) (Fig. 2C), suggesting that *Cyclin B1* expression is not stably entrained to 16-h days. Thus, the significantly lower amplitude of clock oscillations in the 8L:8D cycle might not be enough to stably drive the expression of some indirect clock target genes, such as *Cyclin B1*.

A more complex expression pattern was observed when the cell lines were exposed to a 6L:6D cycle. The core clock gene *Per1* showed a high expression peak in the first LD cycle followed by a small expression peak in the second LD cycle. This pattern was repeated for the whole LD period (Fig. 2D). Although *Per1* expression peaked at every LD cycle, the clearly different amplitude in consecutive cycles suggests that the circadian clock is not actually entrained to a 12-h day. It is likely that the small-amplitude *Per1* peaks are an acute light-driven response, because the bioluminescence increase is observed immediately after the transition from dark to light, while the bioluminescence increase of the high-amplitude peaks starts during the dark phase, indicating an anticipatory response to the light signal characteristic of true entrainment (Fig. 2D). *p21* and *p20* oscillations exhibited a clear 24-h rhythm, supporting this masking idea. However, both waveforms were significantly different from normal, with *p21* expression taking longer to reach peak values, while *p20* expression remained longer at peak levels (Fig. 2D). These changes occurred precisely at the same time of the day as the small peak in *Per1* expression was observed. *Cyclin B1* also showed a 24-h entrainment to the 12-h days (Fig. 2D). It is possible that these results are an example of frequency demultiplication, a characteristic of self-sustaining oscillators that occurs when the period of the entraining cycle is a submultiple of the oscillator’s own period (Pittendrigh, 1981; Roenneberg et al., 2005), leading, in this case, to one expression peak every two 12-h LD cycles, but it is much more likely that a 12-h cycle simply sits outside the range of entrainment for the zebrafish clock. A mathematical perspective on such data might find these observations to be highly predictable. As the driving T-cycle becomes significantly shorter than 24 h and the core oscillator can no longer complete a “normal” molecular oscillation, it is highly likely that a series of period doubling bifurcations would be observed for such an oscillation. It is clear that these data demand a more detailed level of computational analysis in the future.

A very short T-cycle (4L:4D) was necessary to abolish some of the circadian expression rhythms measured in our luminescent cell lines. Under 4L:4D conditions, *Per1* showed extremely low-amplitude rhythms (probably due to an acute light driven response), while *p21, p20*, and *Cyclin B1* exhibited residual oscillations or even arrhythmic expression (Fig. 2E). The clock-controlled genes still showed some expression rhythmicity on the first LD cycles reminiscent of the ones observed in 6L:6D cycles; however, those 24-h rhythms quickly disappeared when the 4L:4D cycle was maintained over several days (Fig. 2E).

The flexibility of the zebrafish circadian clock was tested not only with shorter T-cycles but also with a longer one. Specifically, a 16L:16D cycle led to entrainment in gene expression of all 4 genes with periods matching the 32-h day (Fig. 2F). The core clock, as well as the output genes tested, all entrained well to the long T-32 cycle. Importantly, when the cell lines were transferred to free-running conditions (DD) after exposure to the different T-cycles, expression rhythms with a period slightly greater than 24 h were always observed (Fig. 2). The free-running period varied, in most cases, between 26 h and 27.5 h, which is consistent with the range of free-running periods previously described for individual zebrafish PAC2 cells (24.5-28.3 h) (Carr and Whitmore, 2005). Interestingly, these results suggest that the various T-cycles tested in this study do not cause significant “after effects” on the subsequent free-running period.

Together, these results show that the zebrafish circadian clock is entrained to, at least, a 16-h range of LD cycles (from 8L:8D to 16L:16D). Furthermore, manipulation of LD conditions can accelerate (shorter T-cycles) or retard (longer T-cycles) clock oscillations and, consequently, modify the expression of clock target or downstream genes.

### T-cycle Entrainment Modulates the Timing of Peak Expression of Clock-related Genes

**Figure 3.**
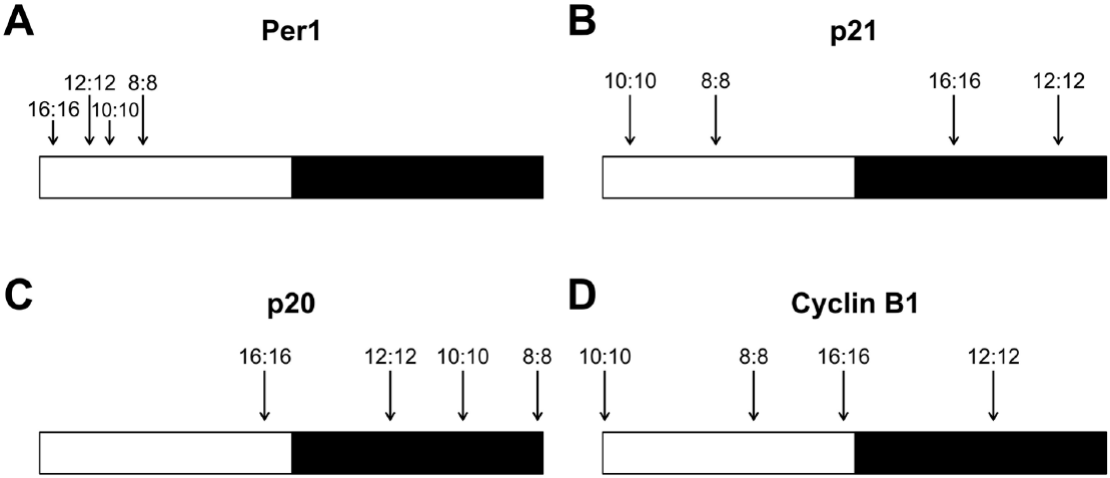
T-cycle entrainment modulates the timing of peak expression of clock-related genes. The peaks of expression of (A) *Per1*, (B) *p21*, (C) *p20*, and (D) *Cyclin B1* are indicated under 16L:16D, 12L:12D, 10L:10D, and 8L:8D cycles. To directly compare the 4 different T-cycles in the same schematic, the relative peak of expression was calculated by dividing the ZT of each expression peak by the respective period length. White and black bars represent light and dark phases, respectively.

The exposure of zebrafish cell lines to different T-cycles also revealed that the peak of expression of rhythmic genes occurs at different relative times of the day in different LD conditions. There is, therefore, a clear change in the phase angle between the clock, clock-outputs, and the LD cycle. In general, shorter T-cycles resulted in a delay of the peak of expression in the relative time of the day, whereas longer T-cycles promoted an advance of the expression peak (Table 1 and Fig. 3). For example, the *p21* expression peak in the normal 12L:12D cycle occurred at the late dark phase; in the shorter T-cycles (10L:10D and 8L:8D), the *p21* expression peak was delayed to the following light period; and in the longer T-cycle (16L:16D), the *p21* expression peak was advanced to the middle of the dark phase (Table 1 and Fig. 3B). Similar changes were observed for the 3 other genes included in our analysis, and when the peaks of expression are ordered from the longest T-cycle (16L:16D) to the shortest T-cycle (8L:8D), the delay in their occurrence relative to the time of day becomes clear (Fig. 3).

**Table 1.** Peak of expression of 4 clock-related genes under different T-cycles.

	Peak of Expression (Relative Time of the Day and ZT)			
	Per1	p21	p20	Cyclin B1
8L:8D	Middle light phase	Middle light phase	Late dark phase	Middle light phase
	3.3 ± 0.25	3.6 ± 0.41	15.8 ± 0.10	4.8 ± 0.69
10L:10D	Early light phase	Early light phase	Late dark phase	Early light phase
	2.8 ± 0.19	1.1 ± 0.36	16.8 ± 0.25	0.1 ± 0.79
12L:12D	Early light phase	Late dark phase	Early dark phase	Middle dark phase
	2.4 ± 0.15	21.7 ± 0.13	16.7 ± 0.18	18.6 ± 0.49
16L:16D	Early light phase	Middle dark phase	Late light phase	Late light phase
	0.9 ± 0.93	22.3 ± 0.44	14.3 ± 0.47	15.3 ± 0.45

Note that for some genes, the peak of expression in ZT is very similar in different T-cycles; however, the relative time of the day can be significantly different because of the variable day length.

Different genes exhibited different degrees of variation in the relative time of the day of their expression peaks: *Per1* showed very little variation, with the peak of expression always occurring in the light phase (Fig. 3A); *Cyclin B1* was particularly sensitive to changes in the period length, leading to peaks of expression occurring at very different times of day (Fig. 3D); *p21* and *p20* showed an intermediate degree of variation when compared with *Per1* and *Cyclin B1* (Fig. 3). Importantly, these changes in gene expression suggest that clock-regulated processes, particularly cell cycle regulation, may occur at different relative times of the day in different LD cycles. This has potentially interesting implications as a method to control the exact daily timing of cell cycle events.

### The Cell Cycle Itself Is Also Entrained to a Wide Range of T-cycles

**Figure 4.**
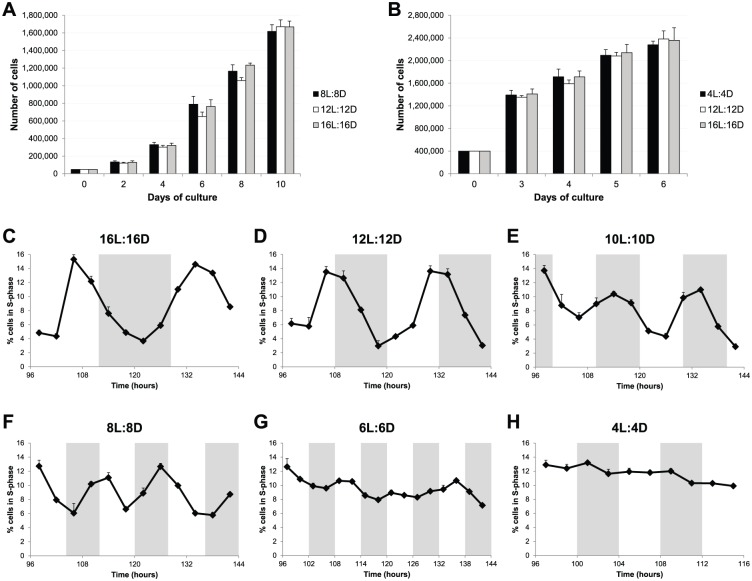
Cell cycle rhythms are also entrained to a wide range of T-cycles. (A) Total number of PAC2 cells over a 10-day period of culture exposed to 8L:8D, 12L:12D, or 16L:16D cycles. (B) Total number of PAC2 cells over a 6-day period of culture exposed to 4L:4D, 12L:12D, or 16L:16D cycles. No statistical difference was found between different T-cycles (1-way ANOVA). (C-H) Percentage of PAC2 cells in S phase (BrdU-positive cells) when exposed to (C) 16L:16D, (D) 12L:12D, (E) 10L:10D, (F) 8L:8D, (G) 6L:6D, or (H) 4L:4D cycles. White and gray backgrounds represent light and dark phases, respectively.

Gene expression of several cell cycle regulators can be modified by a wide range of T-cycles. Do these changes in gene expression lead to significant changes in the rate and timing of cell proliferation? Can cell cycle rhythms observed under normal LD cycles be entrained to new period lengths? To address these questions, we first analyzed cell proliferation under different T-cycles by counting the total number of cells on different days as the experiment progressed. As shown in Figure 4A, we found no significant differences in the total number of cells exposed to 8L:8D, 12L:12D, or 16L:16D cycles for up to 10 days of culture. These LD cycles are able to entrain clock-regulated gene expression, but the global rate of cell proliferation does not seem to be affected.

We have previously reported that clock-regulated cell cycle rhythms in zebrafish cell lines are observed only in cultures approaching confluence (Tamai et al., 2012). Zebrafish cells differ somewhat from their mammalian equivalents in that they do not show strong contact inhibition and continue to divide even at high cell densities. Therefore, we performed similar cell proliferation assays with a higher starting cell density. We compared the shortest T-cycle (4L:4D), where no significant cell cycle gene oscillation was detected, against the normal 12L:12D cycle and the longest T-cycle (16L:16D). Once again, no significant differences in the total number of cells were observed for up to 6 days of culture (Fig. 4B). These results show that different T-cycles, and the gene expression changes that they induce, do not alter the proliferation rate in our zebrafish cell line.

Nonetheless, these results do not inform us about how cell divisions are distributed throughout the different LD cycles and whether clock-controlled cell cycle rhythms are maintained in some or all of the T-cycles. To address these issues, the percentage of cells in S phase was determined, at multiple times of the day under different T-cycles, by BrdU pulsing followed by flow cytometric quantification of BrdU-positive cells. This analysis revealed clear S phase rhythms in 16L:16D, 12L:12D, 10L:10D, and 8L:8D conditions (Fig. 4, C-F). In contrast, in the 6L:6D cycle, no obvious rhythm was observed, although percentage variations were registered during the period analyzed (Fig. 4G); and in the 4L:4D cycle, entry into S phase was arrhythmic (Fig. 4H). Importantly, the LD cycles that exhibited a clear S phase rhythm were the same ones able to perfectly entrain gene expression of clock and clock-output genes. Moreover, *p21* is the predominant clock-controlled G1/S cell cycle inhibitor expressed in zebrafish cell lines (Laranjeiro et al., 2013), and therefore, we would expect it to control S phase entry in the different T-cycles. In fact, the peak of cells in S phase always occurred during the trough of *p21* expression in all the T-cycles tested (Suppl. Fig. S1).

These results show that not only gene expression but also cell cycle regulation can be entrained to a wide range of period lengths (from at least 16-h up to 32-h days). However, these T-cycles do not change the global rate of cell proliferation. Instead, they promote cell cycle progression at the population level to preferentially occur, more or less often depending on the T-cycle, at defined time windows.

### Single Cell Tracking Reveals That Circadian Cell Cycle Timing Occurs via Gating Rather Than a Coupling Mechanism

Our results clearly show that cell cycle progression in zebrafish cell lines can be entrained to a wide range of different T-cycles at the population level. However, a major question remains: How does the circadian clock control cell cycle progression at the single cell level to achieve these population cell cycle rhythms? As described above, one hypothesis is that the circadian clock and the cell cycle are directly coupled, and thus the cell cycle length of individual cells would be the same as (or a multiple of) the circadian period (also known as a phase locking mechanism). The alternative hypothesis is the gating mechanism, in which the circadian clock creates time windows when cell cycle progression is more likely to occur, and therefore the cell cycle length of individual cells does not necessarily have to be different in different T-cycles.

**Figure 5.**
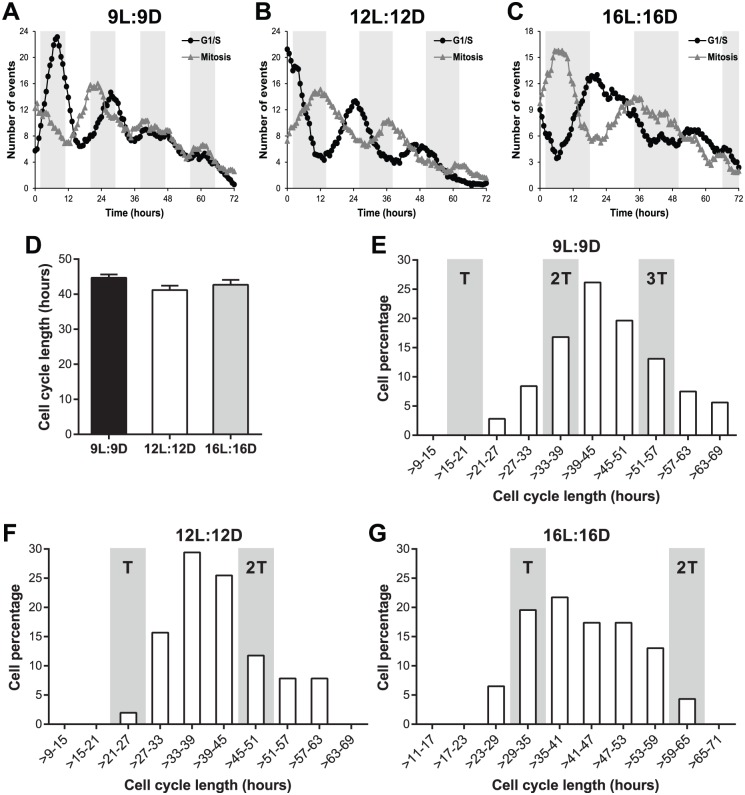
Circadian cell cycle timing occurs via a gating mechanism. (A-C) Number of G1/S transitions and mitoses observed in a zebrafish FUCCI cell line exposed to (A) 9L:9D, (B) 12L:12D, or (C) 16L:16D cycles. G1/S transition was defined as the loss of the nuclear Kusabira-Orange fluorescence, whereas mitosis was defined as the splitting of the nuclear Azami-Green fluorescence into 2 nuclei. Data presented as a 9-point moving average. White and gray backgrounds represent light and dark phases, respectively. (D) Length of the whole cell cycle measured in individual FUCCI cells exposed to 3 different T-cycles. Whole cell cycle length was defined as the time between 2 consecutive G1/S transitions or 2 consecutive mitoses. No statistical difference was found between different T-cycles (1-way ANOVA). (E-G) Distribution of whole cell cycle lengths of FUCCI cells exposed to (E) 9L:9D, (F) 12L:12D, or (G) 16L:16D cycles into 6-h duration bins. Gray columns indicate the cell cycle length matching the respective T-cycle period (T) or a multiple of the T-cycle period (2T and 3T). Data presented in D-G were obtained by tracking single cells in the dataset presented in A-C.

To address this question, we used a zebrafish FUCCI (fluorescent ubiquitination-based cell cycle indicator) (Sakaue-Sawano et al., 2008; Sugiyama et al., 2009) cell line (Downey et al., 2011) that allows for quantitation of the duration of cell cycle phases at the single cell level. We exposed the FUCCI cell line to a short T-cycle (9L:9D), a normal LD cycle (12L:12D), and a long T-cycle (16L:16D), during which time-lapse imaging was performed every 40 min for 72 h. As shown in Figure 5 (A-C), cell cycle rhythms (for both G1/S transition and mitosis) at the population level were observed in all 3 LD cycles, proving that our experimental setup for time-lapse recording did not significantly affect circadian cell cycle entrainment. Importantly, single-cell tracking revealed that the average length of the entire cell cycle is identical for 9L:9D, 12L:12D, and 16L:16D cycles (between 41 and 45 h) (Fig. 5D). Moreover, a detailed distribution analysis showed that there was no enrichment for whole cell cycle lengths at the respective T-cycle period (or a multiple of the T-cycle period) that the cells were exposed to (Fig. 5, E-G). For example, when exposed to a 24-h circadian period (12L:12D), most cells exhibited an entire cell cycle length different from 24 or 48 h (Fig. 5F). Single-cell tracking also revealed no significant differences in G1 length between the 3 T-cycles and a slight reduction in S/G2/M length in 9L:9D and 16L:16D cycles when compared with the normal 12L:12D cycle (Suppl. Fig. S2A). Distribution analyses showed that G1 length varied widely (Suppl. Fig. S2B), whereas S/G2/M duration was mostly fixed to between 9 and 21 h for all T-cycles (Suppl. Fig. S2C). This supports the idea that the majority of clock-cell cycle regulation occurs at the G1-S transition and fits with the key role played by p21 in this process.

These results show that the population cell cycle rhythms observed in different T-cycles are achieved through a gating mechanism and not a coupling mechanism, given that there is no change in the average cell cycle length of individual cells. Instead of directly controlling cell cycle length, the circadian clock modulates the expression of several cell cycle genes, which ultimately results in preferential time windows, or gates, for timed cell cycle progression.

## Discussion

The circadian clock can be entrained by different environmental cues, but for most organisms, light is the major synchronizing signal (zeitgeber). The zebrafish has become a useful circadian model organism for entrainment studies due to the highly decentralized nature of its circadian system, in particular, the direct light entrainability of its cells and tissues. In vitro experiments to assess both the speed and range of entrainment of vertebrate circadian clocks are, in general, extremely difficult or even impossible to perform due to the absence of a reliable or relevant synchronizing signal to the cellular clock. Induction of circadian oscillations in mammalian cell lines can be achieved by serum shock (Balsalobre et al., 1998) or other drug treatments (e.g., forskolin and dexamethasone) (Akashi and Nishida, 2000; Balsalobre et al., 2000; Yagita and Okamura, 2000); however, these signals are useful only to initiate free-running oscillations (~24 h) of the circadian clock. In addition, such drug treatments could have direct effects on clock outputs, such as the cell cycle, independent of the circadian clock. In contrast, the direct light-responsiveness of zebrafish cells allows for the direct synchronization or entrainment of the cellular clock by external LD cycles (Whitmore et al., 2000). This unique characteristic among vertebrates has allowed us to assess both the speed and the limits of entrainment of the zebrafish circadian clock to external light signals in vitro and, furthermore, provides us with a useful tool to assess the reentrainment of downstream clock processes.

Reentrainment experiments, by inverting the LD cycle given to cells in culture, revealed a remarkably quick adaptation of the cell cycle to the new lighting regime. An antiphasic S phase rhythm was established within 36 h of DL exposure. Cell cycle reentrainment begins by a small, but measureable increase in the number of cells entering S phase from G1, followed by a decline to low, antiphasic entrained levels. *p21* expression levels match this S phase reentrainment profile precisely, with an initial decline followed by a fully reset peak expression level within 36 h. This extremely rapid change in *p21* expression is not surprising given its direct transcriptional regulation by the circadian clock through multiple E-box elements within its regulatory DNA/promoter region (Laranjeiro et al., 2013). *Cyclin B1* expression reentrains much more slowly than *p21*, taking an additional circadian cycle to fully reset to the new DL cycle. Although *cyclin B1* rhythms are believed to be critical for timing mitotic events, there is no evidence that the clock directly regulates its expression, in contrast to *p21*. So, the slower resetting of *cyclin B1* is perhaps not surprising. Furthermore, it is plausible that the rhythmic expression of *Cyclin B1* might be the consequence of cell cycle rhythmicity, rather than a cause of it. In such a scenario, the clock primarily couples to the cell cycle via *p21* and S phase resetting, with mitosis following later on. Although we have approached the issue of clock-cell cycle entrainment in this article as a unidirectional coupling process, it is of course possible that the cell cycle itself could influence clock oscillations. Such bidirectional coupling has been described in a number of clock systems, including NIH3T3 cells, where, in fact, the impact of the cell cycle on the clock appears to be the dominant synchronizing signal (Bieler et al., 2014). Although we cannot exclude this possibility in our zebrafish cells, we equally have no evidence to date that it occurs. The direct light-entrainability of our cell system and the robustness of the cellular clock lead us to believe that the dominant coupling signal in this system is from the clock to the cell cycle, rather than the converse.

To explore the range of entrainment of both the zebrafish circadian clock and the cell cycle, we exposed cells to a wide range of period lengths or T-cycles. We have shown that the zebrafish circadian clock can be entrained to LD cycle lengths from at least 16 to 32 h, and this entrainment leads to significant changes in gene expression of core clock and clock target genes. Period lengths of 12 h or less fall outside this range of entrainment and lead to either 24-h output rhythms or arrhythmicity. Not only can the molecular core clock entrain to this wide range of period lengths between 16 and 32 h, but so can *p21* and *cyclin B1* expression. The molecular cell cycle rhythm periods perfectly match the entraining LD cycle lengths, although *cyclin B1* entrainment begins to fail at 8L:8D (T = 16 h), before *p21* fails to entrain. Perhaps more important, actual cell cycle progression also entrains to these T-cycles and matches the underlying gene expression rhythms. Clear rhythms in S phase timing can be seen on T-cycles ranging again from 16 to 32 h. These cell cycle oscillations are lost when cells are placed on T-cycles outside of the range of entrainment (i.e., T = 12 and T = 8 h). Our BrdU pulse experiments clearly showed that within the limits of entrainment, cell cycle progression is regulated to occur at specific times of the day.

Both the actual cell cycle and cell cycle gene expression analyses in luminescent zebrafish cell lines revealed that entrainment to non-24-h cycles leads to a change in the relative time of the day of both S phase and the peak of gene expression (i.e., a change in phase angle). The expression peak was delayed in shorter T-cycles and advanced in longer T-cycles, which is consistent with entrainment theory on how T-cycles affect clock oscillations (Hoffmann, 1963; Merrow et al., 2005). This change in phase angle is quite dramatic, with S phase moving from the middle of the night on a T = 20 to early/mid-day on a T = 32. As such, T-cycles can be a useful tool for shifting the precise timing of cell cycle events and a useful method in helping to time pharmacological anticancer treatments. At the level of gene expression, it is clear that some genes were more sensitive to period length changes than others. These differences are likely correlated to the different levels of clock regulation: *Per1* is a core clock gene and was only slightly affected, *p21* and *p20* are direct clock targets and were moderately affected, and *Cyclin B1* is an indirect clock target and was highly affected. Therefore, small variations in the core clock mechanism can have a major impact on output processes, such as cell cycle timing, probably due to the accumulation of small differences in downstream pathways. From these results, it appears that the cell cycle is directly coupled, 1:1 to the period of the circadian pacemaker, at least at the population level, and can change velocity over a remarkable range from a rapid cell cycle of 16 h to a much slower one of 32 h. If the cell cycle speed in zebrafish could be doubled, as appears from the above data, then one would expect to see quite a remarkable change in cell proliferation rates depending on the LD cycle length. However, our results from counting cells on different T-cycles for many days does not support this conclusion. In fact, the growth curves are remarkably similar regardless of the entraining cycle length and raise doubts about the nature of the 1:1 coupling we apparently observe in cell populations.

To attempt to resolve this apparent contradiction, single cell tracking of cell cycle progression using zebrafish FUCCI cells was essential. Although single cell analysis of the cell cycle has been performed in mammalian cell lines, in none of these studies was it possible to manipulate the core clock mechanism, as in zebrafish, by entraining the cell cycle to different period lengths. Analysis of cell cycle length using the FUCCI reporter cells shows that the average cell cycle length of individual cells was unaffected by the different T-cycles. In fact, the cell cycle length of these zebrafish cell lines is quite long, at around 41 to 45 h, but is constant regardless of the driving entrainment cycle. It is clear that unlike in mammalian fibroblasts, there is not a 1:1 coupling of clock to cell cycle in these zebrafish cultures. Considering the naturally long length of the zebrafish cell cycle, perhaps it is not so surprising that a direct coupling mechanism is not at play. Consequently, circadian-cell cycle rhythms are achieved by cell cycle progression at preferential time windows, a gating mechanism, rather than a direct coupling between the 2 oscillators. In zebrafish cells, the circadian oscillator entrains a gate or window during which cell cycle progression can occur. As the range of clock entrainment in zebrafish is very wide, this also means that at the population level, one can measure a remarkably wide range of cell cycle periods.

The temporal correlation between the low point of *p21* expression and maximum number of cells present in S phase is maintained at all of the T-cycles examined, except when outside the range of clock entrainment (Suppl. Fig. S1). Cell cycle gating, through the use of clock-controlled checkpoints, is clearly the mechanism used in zebrafish to regulate daily cell cycle events. What is the nature of the gate that regulates cell cycle timing in these cells? We know from previous studies that both *p20* and *p21* are critical regulators of S phase progression in zebrafish (Laranjeiro et al., 2013). Equally, the expression of both of these genes is under robust clock control. We believe that both the timing and the duration of this *p21* expression window define the gate, at least regarding the timing of S phase events. When *p21* expression levels fall, then the inhibition of progression from G1 to S phase is reduced, and cells are more likely to enter S phase and begin DNA replication. However, this is a probabilistic event, and all other essential requirements of the cell cycle must be met. Clearly, we need to confirm this hypothesis in the future with a detailed analysis of p21 protein levels in individual cells, but the current model is that the clock-controlled expression of *p21* does, in fact, define the S phase cell cycle gate.

It is our conclusion that cell cycle entrainment occurs through a gating process, rather than a phase locking mechanism in which the length of the cell cycle period matches the length of the driving light-dark cycle. This does raise, however, the interesting possibility that entrainment of the core clock mechanism itself could be set this way, through a gating mechanism, rather than through a classic period entrainment mechanism. In this way, light might establish a “window,” or stochastic “entrainment” in individual cells, which then appears as a perfect 24-h oscillation at the cell population level. Although we have not yet successfully imaged clock gene expression in single cells under differing T-cycles, our previous imaging studies have shown approximate 24-h *period1-luciferase* oscillations, following entrainment to a 24-h light-dark cycle (Carr and Whitmore, 2005). The simplest interpretation of these data is that the single cell clock is entraining in a classic, phase-locked manner to the driving light-dark cycle. We do not see a very wide range of individual cell circadian periods, which would require a gating event for effective entrainment, although it must be said that there is considerable noise in the data at the single cell level. In addition, the zebrafish cellular clock can entrain to skeleton photoperiods in culture, in a manner predicted by classic entrainment models (Tamai et al., 2007). The duration of light exposure does not have a massive impact on entrainment of phase or period. Consequently, we believe that the central cellular clock mechanism is most likely set through the expected alterations in its period length to match the duration of the external entraining cycle. This is in contrast to the cellular process of timed cell division, which we believe is regulated through a downstream, clock-controlled gating mechanism.

## Supplementary Material

Supplementary material

Supplementary material
