# Supplementary material for: Circadian Clock Synchronization of the Cell Cycle in Zebrafish Occurs through a Gating Mechanism Rather Than a Period-phase Locking Process
Source: J Biol Rhythms. 2018 Feb 14;33(2):137–50. doi: 10.1177/0748730418755583 (PMC5944076; doi:10.1177/0748730418755583)
Supplement: Supplementary material [file supplementary_material1.pdf]

Supplementary Material

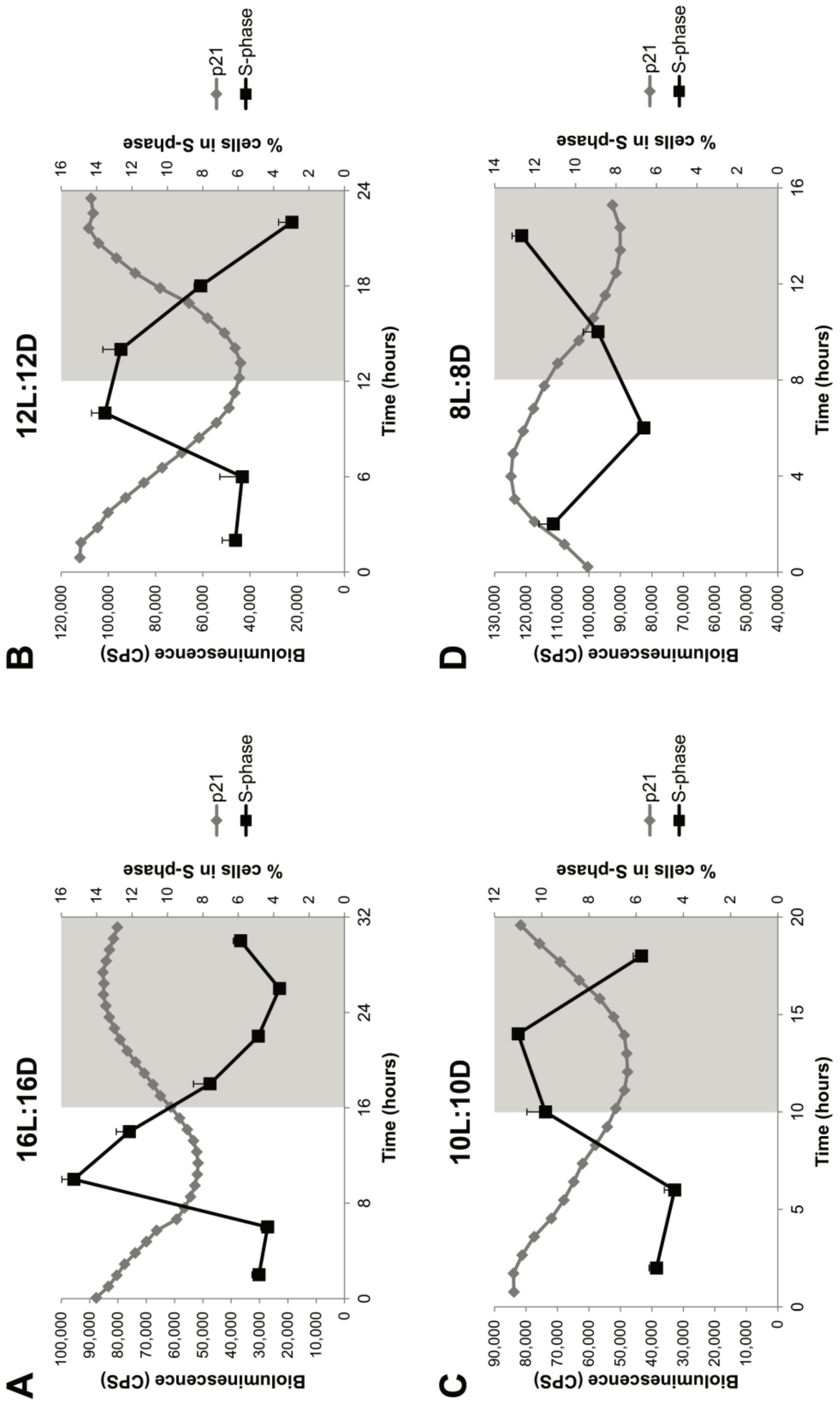

Figure S1. p21 controls S phase entry in zebrafish cell lines under different T-cycles. (**A-D**) Bioluminescent trace of *p21-luciferase* cell line plotted against the percentage of cells in S phase exposed to 16L:16D (**A**), 12L:12D (**B**), 10L:10D (**C**), or 8L:8D (**D**) cycles. Data presented in Fig. 2 and Fig. 4 were used to create these graphs (one LD cycle). White and grey backgrounds represent light and dark phases, respectively.
