# Supplementary material for: Circadian Clock Synchronization of the Cell Cycle in Zebrafish Occurs through a Gating Mechanism Rather Than a Period-phase Locking Process
Source: J Biol Rhythms. 2018 Feb 14;33(2):137–50. doi: 10.1177/0748730418755583 (PMC5944076; doi:10.1177/0748730418755583)
Supplement: Supplementary material [file supplementary_material2.pdf]

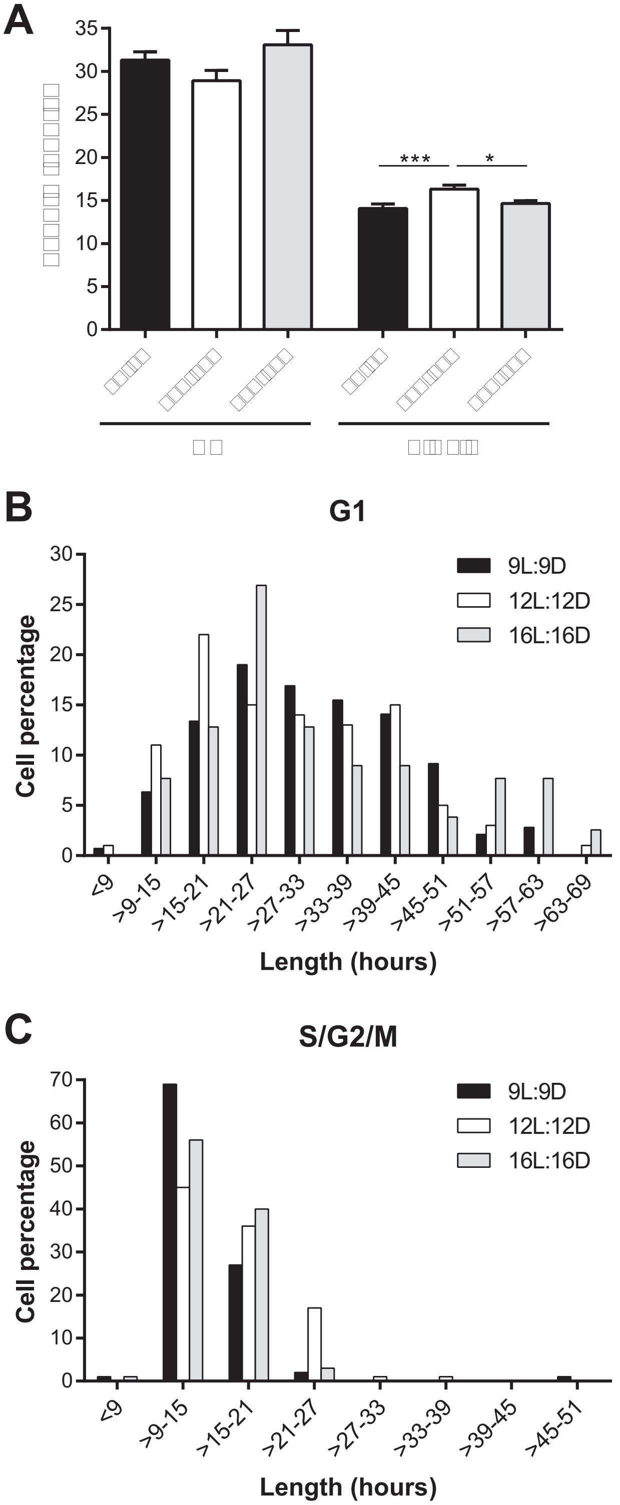

Figure S2. Length of cell cycle phases under different T-cycles. (A) Length of G1 and S/G2/M phases measured in individual FUCCI cells exposed to 9L:9D, 12L:12D, or 16L:16D cycles. G1 and S/G2/M lengths were defined as the time that single cells maintained the Kusabira-Orange fluorescence or the Azami-Green fluorescence, respectively. No statistical difference was found for G1 lengths (one-way ANOVA), whereas statistical differences were found for S/G2/M lengths (one-way ANOVA followed by Tukey's post hoc test). **P*<0.05; ****P*<0.001. (**B-C**) Distribution of G1 (**B**) and S/G2/M (C) phases of FUCCI cells exposed to the three different T-cycles into 6-hour duration bins. Note that for the short and long T-cycles, 95% of the cells exhibited an S/G2/M duration between 9 and 21 hours, whereas for the 12L:12D cycle that proportion was 81% (and 17% between 21 and 27 hours), which justifies the slight reduction in S/G2/M length in 9L:9D and 16L:16D cycles presented in (A). Data presented were obtained by tracking single cells in the dataset presented in Fig. 5A-C.
